# Supplementary material for: Morphological and Genetics Support for a Hitherto Undescribed Spotted Cat Species (Genus Leopardus; Felidae, Carnivora) from the Southern Colombian Andes
Source: Genes (Basel). 2023 Jun 15;14(6):1266. doi: 10.3390/genes14061266 (PMC10298493; doi:10.3390/genes14061266)
Supplement: Supplementary file 1 [file genes-14-01266-s001.zip › genes-2358502-supplementary.pdf]

Table S1. Tigrina-like specimens and other *Leopardus* individuals analyzed for two different mitochondrial data sets (1- mt*ND5*; 2- mitogenomes**, including mt*Cyt-b* gene). mt** = mitochondrial. PUJ = Mammal collection of the Laboratory of Molecular Population Genetics and Evolutionary Biology at the Pontificia Universidad Javeriana (Bogotá, Colombia). IvH = Mammal collection of the Instituto von Humboldt (Villa de Leyva, Colombia).

Taxon	Country	Precise origin of each specimen	mt data set analysed and accession voucher numbers (ID)	References and GenBank accession numbers
***L. tigrinus* and a new little spotted cat species**	Costa Rica	Arenal Cordillera Volcánica Central	1, 2<br>PUJ ID: lti81	This work<br>GenBank: MG230227.1
	Costa Rica	Guanacaste	1, 2<br>PUJ ID: lti82	This work<br>GenBank: MG230228.1
	Venezuela	Mesa Bolivar, Mérida	1<br>PUJ ID: lti29	This work<br>GenBank: MG230207.1
	Colombia	Valdivia, Antioquia	1, 2<br>PUJ ID: lti68	This work<br>GenBank: MG230196.1
	Colombia	Valdivia, Antioquia	1<br>PUJ ID: lti38	This work<br>GenBank: MG230202.1
	Colombia	Caucasia, Antioquia	1, 2<br>PUJ ID: lti53	This work<br>GenBank: MG230198.1
	Colombia	Caucasia, Antioquia	1<br>PUJ ID: lti25	This work<br>GenBank: MG230210.1
	Colombia	Sonsón, Antioquia	1<br>PUJ ID: lti12	This work<br>GenBank: MG230214.1
	Colombia	Barbosa, Antioquia	1<br>PUJ ID: lti3	This work<br>GenBank: MG230216.1
	Colombia	Nevado del Ruiz, Caldas	1, 2<br>PUJ ID: lti8	This work<br>GenBank: MG230203.1
	Colombia	La Virginia, Risaralda	1, 2<br>PUJ ID: lti53	This work<br>GenBank: MG230204.1
	Colombia	Apia, Risaralda	1, 2<br>PUJ ID: lti61	This work<br>GenBank: MG230197.1
	Colombia	Circasia, Quindio	1<br>PUJ ID: lti35	This work<br>GenBank: MG230212.1
	Colombia	Tunja, Boyacá	1<br>PUJ ID: lti1	This work<br>GenBank: MG230218.1
	Colombia	Chita, Boyacá	1, 2<br>PUJ ID: lti2	This work<br>GenBank: MG230219.1
	Colombia	Coper, Boyacá	1<br>PUJ ID: lti7	This work<br>GenBank: MG230220.1
	Colombia	Chingaza National Park, Cundinamarca	1<br>PUJ ID: lti19	This work<br>GenBank: MG230201.1
	Colombia	Chingaza National Park, Cundinamarca	1<br>PUJ ID: lti33	This work<br>GenBank: MG230205.1
	Colombia	El Refugio, La Macarena, Meta	1<br>PUJ ID: lti14	This work<br>GenBank: MG230213.1
	Colombia	Pitalito, Huila	1<br>PUJ ID: lti28	This work<br>GenBank: MG230208.1
	Colombia	San Agustín, Huila	1<br>PUJ ID: lti30	This work<br>GenBank: MG230206.1
	Colombia	La Plata, Huila	1<br>PUJ ID: lti13	This work<br>GenBank: MG230222.1
	Colombia	Jamundí, Valle del Cauca	1<br>PUJ ID: lti40	This work<br>GenBank: MG230200.1
	Colombia	Ginebra, Valle del	1	This work

		Cauca	PUJ ID: lti9	GenBank: MG230221.1
	Colombia	Florencia, Caquetá	1<br>PUJ ID: lti11	This work<br>GenBank: MG230215.1
	Colombia	Galera Volcano, Nariño	1, 2<br>IvH ID: 5857	This work<br>GenBank: MG230232.1
	Ecuador	Concepción, Esmeraldas	1<br>PUJ ID: lti26	This work<br>GenBank: MG230225.1
	Ecuador	Intag, Imbabura	1, 2<br>PUJ ID: lti42	This work<br>GenBank: MG230224.1
	Ecuador	Papallacta, Napo	1<br>PUJ ID: lti43	This work<br>GenBank: MG230199.1
	Ecuador	Pajuli, Cotopaxi	1, 2<br>PUJ ID: lti22	This work<br>GenBank: MG230223.1
	Peru	Lamas, San Martín	1<br>PUJ ID: lti21	This work<br>GenBank: MG230211.1
	Bolivia	Cotapata National Park, Yungas, La Paz	1<br>PUJ ID: lti27	This work<br>GenBank: MG230209.1
	Bolivia	Cotapata National Park, Yungas, La Paz	1<br>PUJ ID: lti36	This work<br>GenBank: MG230217.1
	Argentina	Border Salta and Tucumán	1<br>PUJ ID: lti15	This work<br>GenBank: MG230226.1
	Argentina	Puerto Iguazú, Misiones	1, 2<br>PUJ ID: lti16	This work<br>GenBank: MG230229.1
	Argentina	Puerto Iguazú, Misiones	1, 2<br>PUJ ID: lti17	This work<br>GenBank: MG230230.1
	Argentina	San Pedro, Misiones	1, 2<br>PUJ ID: lti18	This work<br>GenBank: MG230231.1
	Brazil	Dois Vizinhos, Paraná	1, 2<br>PUJ ID: lti83	This work<br>GenBank: MG230233.1
	Brazil	Dois Vizinhos, Paraná	1, 2<br>PUJ ID: lti84	This work<br>GenBank: MG230234.1
	Brazil	Unknown origin	1	GenBank (Johnson & O'Brien 1997)
	Brazil	Unknown origin	1, 2	GenBank (Li et al. 2016)
L. colocola	Unknown origin	Unknown origin *colocola*	1	GenBank (Johnson & O'Brien 1997)
	Brazil	Unknown origin *braccatus*	1, 2	GenBank (Li et al. 2016)
	Brazil	Unknown origin *braccatus*	1, 2	GenBank (Li et al. 2016)
L. geoffroyi	Bolivia	Puerto Suárez, Santa Cruz	1, 2<br>PUJ ID: lge08	This work<br>GenBank: MG230235.1
	Argentina	Unknown origin	1	GenBank (Johnson & O'Brien 1997)
	Argentina	Unknown origin	1, 2	GenBank (Li et al 2016)
	Argentina	Unknown origin	1, 2	GenBank (Li et al. 2016)
L. guigna	Chile	Unknown origin	1	GenBank (Johnson & O'Brien 1997)
	Chile	Unknown origin	1, 2	GenBank (Li et al. 2016)
	Chile	Unknown origin	1, 2	GenBank (Li et al. 2016)
	Chile	Unknown origin	1	GenBank (Napolitano et al. 2008)
	Chile	Unknown origin	1	GenBank (Napolitano et al. 2008)
L. jacobita	Bolivia	La Paz	1, 2<br>PUJ ID: lja14	This work<br>GenBank: MG230236.1
	Bolivia	Cochabamba	1, 2<br>PUJ ID: lja21	This work<br>GenBank: MG230237.1
	Bolivia	Unknown origin	1, 2	GenBank (Li et al. 2016)

	Bolivia	Unknown origin	1, 2	GenBank (Li et al. 2016)
L. pardalis	Colombia	Guainía River, Guainía	1, 2<br>PUJ ID: lpa35	This work<br>GenBank: MG230239.1
	Colombia	San Juan Socó, Loretoyaku River, Amazonas	1, 2<br>PUJ ID: lpa67	This work<br>GenBank: MG230240.1
	Peru	Floresta, Alto Uyacali	1, 2<br>PUJ ID: lpa104	This work<br>GenBank: MG230241.1
	Brazil	Humaitá, Madeira River, Amazonas	1, 2<br>PUJ ID: lpa685	This work<br>GenBank: MG230242.1
	Brazil	Negro River, Amazonas	1<br>PUJ ID: lpa77	This work<br>GenBank: MG230238.1
	Unknown origin	Unknown origin	1, 2	GenBank (Li et al. 2016)
L. wiedii	Colombia	Chigorodó, Antioquia	1, 2<br>PUJ ID: lwi111	This work<br>GenBank: MG230244.1
	Colombia	Leticia, Amazonas	1, 2<br>PUJ ID: lwi95	This work<br>GenBank: MG230245.1
	Ecuador	Tena, Napo	1, 2<br>PUJ ID: lwi118	This work<br>GenBank: MG230243.1
H. yagouaroundi	Guatemala	Unknown origin	1	GenBank (Johnson & O'Brien 1997)
	Colombia	Puerto Escondido, Córdoba	1, 2<br>PUJ ID: pya20	This work<br>GenBank: MG230249.1
	Ecuador	Sarayacu, Pastaza	1, 2<br>PUJ ID: pya77	This work<br>GenBank: MG230248.1
	Peru	Tingo Maria, Huánuco	1, 2<br>PUJ ID: pya22	This work<br>GenBank: MG230250.1
	Unknown origin	Unknown origin	1, 2	GenBank (Li et al. 2016)
	Unknown origin	Unknown origin	1, 2	GenBank (Li et al. 2016)
F. catus	Colombia	Bogotá	1, 2<br>PUJ ID: fca1	This work<br>GenBank: MG230246.1
	Colombia	Bogotá	1, 2<br>PUJ ID: fca2	This work<br>GenBank: MG230247.1
	Colombia	Bogotá	1<br>PUJ ID: fca3	This work<br>GenBank: MG230251.1

Table S2. Mitochondrial and microsatellite primers employed in this work. (**A**) The Long-range amplification mitochondrial primers used. (**B**) Nuclear microsatellite primers used.

(A)

Primer name	Primer sequence (5' to 3')	Amplicon sizes (bp)	Genes contained in each amplicon
LT1F<br>LT1R	ATGACTAATCAGCCCATGAT<br>AAGCATCCCACCTCAAACAT	5,011	*Control region, tRNA-Phe, 12s rRNA, tRNA-Val, 16s rRNA, tRNA-Leu, ND1*
LT2F<br>LT2R	AAGAAATATGTCTGACAAAA<br>TGATCTGCATCTATACTGA	4,015	*tRNA-Ile, tRNA-Gln, tRNA-Met, ND2, tRNA-Trp, tRNA-Ala, tRNA-Asn, tRNA-Cys, tRNA-Tyr, COI, tRNA-Ser, tRNA-Asp* and *COII*
LT3F<br>LT3R	CATTAAGAAGCTAAATAAGC<br>GTGCAACTCCAAATAAAAGT	4,050	*tRNA-Lys, ATP8, ATP6, COIII, tRNA-Gly, ND3, tRNA-Arg, ND4L, ND4, tRNA-His, tRNA-Ser* and *tRNA-Leu*
LT4F<br>LT4R	TAAAAGTAATAAACCTATTT<br>TGGGACATCTCGATGGACTA	3,680	*ND5, ND6, tRNA-Glu, Cyt-b, tRNA-Thr* and

			tRNA-Pro

(B)

Fca08: ACTGTAAATTTCTGAGCTGGCC
TGACAGACTGTTCTGGGTATGG

Fca43: GAGCCACCCTAGCACATATACC
AGACGGGATTGCATGAAAAG

Fca45: TGAAGAAAAGAATCAGGCTGTG
GTATGAGCATCTCTGTGTTCGTG

Fca96: CACGCCAAACTCTATGCTGA
CAATGTGCCGTCCAAGAAC

Fca126: GCCCCTGATACCCTGAATG
CTATCCTTGCTGGCTGAAGG

Fca225: CTGTCTCCCTCCTGTTCCTG
TACCCACAGAACCTTCCTGC
